# Species Detection and Identification in Sexual Organisms Using Population Genetic Theory and DNA Sequences

**DOI:** 10.1371/journal.pone.0052544

**Published:** 2013-01-04

**Authors:** C. William Birky

**Affiliations:** Department of Ecology and Evolutionary Biology, The University of Arizona, Tucson, Arizona, United States of America; Montreal Botanical Garden, Canada

## Abstract

Phylogenetic trees of DNA sequences of a group of specimens may include clades of two kinds: those produced by stochastic processes (random genetic drift) within a species, and clades that represent different species. The ratio of the mean pairwise sequence difference between a pair of clades (K) to the mean pairwise sequence difference within a clade (θ) can be used to determine whether the clades are samples from different species (K/θ≥4) or the same species (K/θ<4) with probability ≥0.95. Previously I applied this criterion to delimit species of asexual organisms. Here I use data from the literature to show how it can also be applied to delimit sexual species using four groups of sexual organisms as examples: ravens, spotted leopards, sea butterflies, and liverworts. Mitochondrial or chloroplast genes are used because these segregate earlier during speciation than most nuclear genes and hence detect earlier stages of speciation. In several cases the K/θ ratio was greater than 4, confirming the original authors' intuition that the clades were sufficiently different to be assigned to different species. But the K/θ ratio split each of two liverwort species into two evolutionary species, and showed that support for the distinction between the common and Chihuahuan raven species is weak. I also discuss some possible sources of error in using the K/θ ratio; the most significant one would be cases where males migrate between different populations but females do not, making the use of maternally inherited organelle genes problematic. The K/θ ratio must be used with some caution, like all other methods for species delimitation. Nevertheless, it is a simple theory-based quantitative method for using DNA sequences to make rigorous decisions about species delimitation in sexual as well as asexual eukaryotes.

## Introduction

One of the most general and basic features of the biological world is that it is discontinuous, divided up into clusters of individual organisms that are very similar to each other and very different from individuals in other clusters. Moreover, members of a cluster of sexual organisms interbreed mainly or exclusively with other members of the same cluster. These are probably the original observations of what we call species, and are widely shared among persons and cultures [1]. Species can be seen as clusters, not only with respect to their visible phenotypes, but also in their physiology and biochemistry, down to the level of DNA sequences. As a result, species have been widely accepted as fundamental units of life in areas of biology ranging from molecular genetics through population genetics to ecology.

Meanwhile, systematists have struggled with the responsibility of actually defining species. Darwin himself emphasized the occurrence of intermediate forms between varities and the difficulty distinguishing varieties from species; these intermediate forms were an important part of his argument for evolution. But he also provided the key to distinguishing species from varieties when he wrote “Hereafter we shall be compelled to acknowledge that the only distinction between species and well-marked varieties is, that the latter are … connected at the present day by intermediate gradations, whereas species were formerly thus connected. Hence, without rejecting the consideration of the present existence of intermediate gradations between any two forms, we shall be led to weigh more carefully and to value higher the actual amount of difference between them” [2]. In *The Descent of Man, and Selection in Relation to Sex* [3] Darwin wrote that “the complete absence, in a well-investigated region, of varieties linking together any two closely-allies forms, is probably the most important of all the criterions of their specific distinctness…”

The problem addressed in this paper is how to use DNA sequences to weigh and value the differences between species and compare them to differences within species, in order to determine when intermediate gradations are in fact missing, and thereby to delimit species. I will show how to distinguish between clusters that are different species and those that are samples from different populations within a single species. The focus is on sexual organisms, extending the theory and methods previously applied to asexual organisms [4], [5], [6], [7]. Outcrossing sexual organisms pose an additional problem not usually encountered with asexuals: once speciation has begun to split one species into two, different recombining genes complete their segregation into two populations at different rates. Fortunately, this problem can be largely circumvented by using organelle genes to detect speciation, because the organelle genome is usually not affected by recombination and achieves reciprocal monophyly in about 1/4 the time of the average nuclear gene in diploid sexual organisms [8]. Consequently we will focus on the use of organelle genes to delimit species.

As a model of what species are, I use the evolutionary genetic species concept (hereafter, EGSC) in which species are inclusive populations that are evolving independently of each other, either because they are reproductively isolated, or because they are separated by environmental or physical barriers, or both [6]. Evolutionary genetic species are independent arenas for mutation, random drift, and natural selection. The EGSC was inspired by the evolutionary species concept (EvSC) [9], [10] which requires that populations must be evolving independently now *and* continue to evolve independently in the future in order to be counted as different species. But this is essentially unknowable. If species are separated physically but have not yet evolved genetically-determined reproductive isolation, there is a possibility that in the future their ranges or habitats may become sympatric and the species would fuse or one would replace the other. Such secondary sympatry is especially likely today as humans transport species across boundaries that the species were formerly unable to cross, or cause climatic changes that result in wholesale species redistribution. Even if two populations have evolved reproductive isolation mechanisms, it is theoretically possible for these to be lost, especially if the reproductive isolation is maintained by only one or a few genetic loci. These are possible outcomes but can rarely be predicted with any confidence.

Given the EGSC, the problem of species delimitation is to identify independently evolving populations based on DNA sequences from individual organisms. Although it would be nice to have, for every one of the species, the kind of in-depth knowledge of the organism that is often produced by systematists for a few species, this is manifestly impractical for all of the estimated 5 to 10 million, or possibly 100 million, species [11]. It is often more practical to obtain DNA sequences of a sample of individuals from a group and delimit species using these sequences. Moreover, environmental sequencing is producing a wealth of sequence data from organisms that are hard to isolate and examine as individuals because of their small size or cryptic appearance, or because they occupy extreme environments; methods such as the one given here can be used to assign these environmental sequences to species, allowing sequence diversity to be partitioned into the diversity of species and of genes within species.

**Figure 1:**
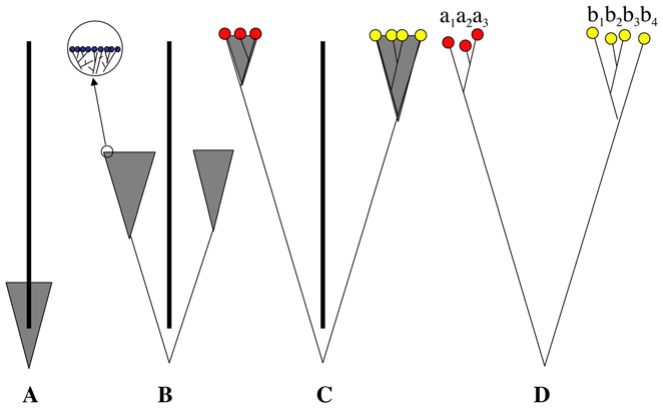
Models of successive times after speciation (A–C) with sampling at stage C (D). The bases of the inverted shaded isosceles triangles in A–C represent populations with effective size N_e_ individuals, while the vertex represent the most recent common ancestor. The altitude of the triangles represents coalescent time = 2N_e_ generations. The vertical bar represents a barrier to gene flow that splits the population in two. The inset in B shows some of the genes (black circles) and their relationships. Red and yellow circles in C and D are samples of genes. Nucleotide diversities π of the two species are estimated by the mean sequence differences a_i_a_j_ and b_i_bj; θ is a function of π and the sample sizes. K is the mean of the sequence differences a_i_b_i_, corrected for multiple hits.

Previously [4], [5], [6], [7] my students and I described a species criterion, the K/θ method, which applies population genetic theory to DNA sequences in order to determine if the sequences came from one, two, or more species. Here K is the average sequence difference between individuals in different species, corrected for multiple hits, and θ is an estimate of the average sequence difference between individuals in the same species. The principle behind the method is illustrated in Figure 1, which shows a single species splitting into two independently evolving populations that gradually diverge over time. The problem is to distinguish the deep, long-lasting gaps between clades of independently evolving populations from the shallow gaps separating clades formed within a single species by stochastic changes in gene frequencies (random genetic drift). Moreover, this must be done using very small samples of specimens from large populations. The K/θ method differs from most previous species criteria in two ways: it is explicitly based on population genetic theory, and it recognizes that species delimitation is based on samples of individuals. It is conceptually similar to the Generalized Mixed Yule Coalescent (GMYC) method [12] which is based on coalescent theory and usually finds the same species [5], [13]. The K/θ method has the following steps:

1. Putative species are first identified as clades in a phylogenetic tree of the DNA sequences. This is best done with methods such as neighbor-joining that produce trees with branch lengths proportional to the number of base pair substitutions along the branches; bootstrapping can be used to identify robust clades. Alternatively, one can use maximum likelihood, Bayesian, or other methods that simultaneously make trees and give support values for clades.
2. To take into account that the clades are identified from a finite sample of individuals, we use a table kindly provided by Noah Rosenberg and based on the theory in [14]. Given the sample sizes from two clades in the tree from step 1 and the K/θ ratio, the table gives the probability that the sequences in the two clades are samples from populations that are reciprocally monophyletic. For organelle genes, θ = 2N_e_μ, where N_e_ is the effective population size of organelle genes and μ is the mutation rate per site per generation. For example, when K≥4N_e_μ, and the clades contain ≥2 individuals each, one can infer that the samples came from two populations that are reciprocally monophyletic with 95% probability. The probability is so high even with such small samples because the probability is conditioned on the samples being reciprocally monophyletic, which was established in the first step.
3. Sister clades in such a tree may be samples from sister species; alternatively they could be subpopulations of a single species that are only partially isolated or transient clusters formed by random drift. To distinguish between these kinds of clades, we note that it takes 4N_e_ generations for 95% of pairs of sister species to become reciprocally monophyletic in an asexual genome [15], [16]. At this point the mean pairwise sequence difference between the species is K = 8N_e_μ. Consequently, if two clades are separated by a sequence difference K≥8N_e_μ, the probability that the clades are independently evolving populations is ≥0.95, while the probability that the clades were formed by random drift withn a single species is ≤0.05. The parameters N_e_ and μ are rarely known and difficult to determine, but can be eliminated by taking the ratio of K to θ = 2 N_e_μ, so that K/θ = 8N_e_μ/2N_e_μ = 4. θ can be calculated from the nucleotide diversity π, which is the mean pairwise sequence difference among members of a clade. Consequently putative species are identified as clades for which K/θ≥4. Note than when the uncorrected difference between clades D is <<1, the probability of multiple changes at a site is negligible and D can be used instead of K.

This procedure addresses the two issues involved in delimiting species. First, are the sample sizes large enough? This statistical issue is addressed in the first and second steps. Second, are the gaps deep enough? This is addressed in the third step by using the K/θ ratio.

We [4] initially referred to this method as the “4× rule” as opposed to the more general term “K/θ ratio.” In principle, if one has independent reason to believe that a pair of sister clades are evolving independently, the K/θ ratio could be used to estimate how long ago speciation occurred.

In our third paper applying K/θ to asexual organisms we noted the method could also be applied to sexual organisms in which it would detect early stages of speciation if mitochondrial genes were used [6]. While the present work was in progress, two other groups followed up on this suggestion by applying the 4× rule to delimit cryptic species in sexual meiobenthic invertebrates [13], [17].

To illustrate the application and utility of this method for sexual organisms, I applied the K/θ method to four sequence data sets from a range of macroorganisms: birds, mammals, molluscs, and liverworts. These examples were chosen from the recent literature to illustrate cases where species were delimited using K/θ with at least 95% statistical confidence, as well as one case with lower confidence that might indicate more recent or possily incomplete speciation. In each case the mitochondrial or chloroplast genes were sequenced at least twice, once in each direction, to assure high quality data. This is important because when a gene is sequenced only once it is difficult or impossible to tell what proportion of sequence differences are due to sequencing errors, as opposed to evolutionary changes. Finally I attempted to make it easier for the reader to follow the argument by choosing cases with only two or three putative species, or from which the sequences from a few potential species could be extracted and analyzed separately. Mitochondrial genes, used for the animals in these examples, are inherited maternally in almost all animals [18] and this was assumed to be true for all of the animals in this study. A significant number of plants show some degree of biparental or even strictly paternal inheritance of chloroplast genes, but chloroplast genes have been shown to be inherited maternally in one species of liverwort [19] and I assumed that is the case for the liverworts in the example considered here.

## Materials and Methods

DNA sequences were downloaded from GenBank, except for liverworts for which Jochen Heinrich provided sequences already aligned. Alignments were checked and sequences trimmed when necessary in MacClade [20], after which PAUP* [21] was used to make phylogenetic trees which were visualized in PAUP* and Dendroscope [22]. When it was necessary to correct sequence differences for multiple hits, evolutionary models were selected with ModelTest [23]. Then pairwise distances were listed in PAUP* and copied into Excel spreadsheets for calculation of K/θ ratios.

Using the K/θ ratio to determine the probability that two samples come from different evolutionary species involves six steps, of which numbers 2–5 are calculated by formulae pasted in the Excel spreadsheet:

1. Find statistically well-supported pairs of sister clades using standard phylogenetic distance methods such as bootstrapped neighbor-joining, maximum likelihood, or Bayesian inference. I use neighbor-joining trees with ≥70% bootstrap support. Each pair is then tested separately, starting at the tips of the tree, to determine the probability that the clades are samples from independently evolving species; this continues until species are found in the following steps.
2. For each clade in a pair, estimate nucleotide diversity π by the mean pairwise difference d between sequences multiplied by the sample size correction n/(n-1), where n is the number of sequences in the clade.
3. For each clade, estimate θ = 2N_e_μ by π/(1–4π/3) [24]. When d = 0, we used a non-zero estimate of π by assuming that one pairwise difference is not zero but instead is 1/L where L is the sequence length; then π = 2/Ln(n-1).
4. Calculate K = mean pairwise sequence difference between the two clades, corrected for multiple hits.
5. Calculate K/θ for the pair of clades. The values of θ for the two clades may differ, in which case I use the larger value of θ to get a conservative estimate of K/θ.
6. Using the K/θ ratio and the numbers n1 and n2 of individuals in the two clades, find the probability that the individuals were sampled from populations that have been evolving independently long enough to become reciprocally monophyletic. This is best done using a table available on request from Noah Rosenberg or me; altenatively the values can be estimated from Figure 6 in [14]. For clades that are members of a non-bifurcating tree such as a polytomy (A, B, C) or ladder ((A,B)C), I compare A and B first, then compare C to whichever one of those is closest to D.

## Results

### Clouded Leopards *Neofelis nebulosa* from Sumatra and Borneo are Different Species from Those on Taiwan and Mainland Borneo

*N. nebulosa* is divided into several subspecies. Two of these, *N. n. nebulosa* from the SE Asia mainland and *N. n. diardi* from Borneo, were proposed to be different species based on their geographic separation, fixed differences in karyotype, and molecular differences at several nuclear and mitochondrial loci [25]. The sequence differences were said to be as great as, or greater than, differences between the five species in the sister genus *Panthera* (lion, tiger, jaguar, leopard, and snow leopard).

Systematics papers sometimes include tables of pairwise sequence differences that can be used to calculate K/θ, eliminating the need to re-analyze the DNA sequences. Table S3A of [25] summarizes the pairwise sequence differences in 139 bp of the mitochondrial *atp8* gene between 3 clouded leopards from Borneo and 64 specimens from the mainland. Using these data I calculated D/θ = 5.65, from which the probability is greater than 0.99 that these are samples from different clades; since D/θ>4, they are from different independently evolving species. (D was 0.0417 which is too low to be much affected by multiple hits, making it reasonable to use D instead of K). Note that π, and hence θ, is normally calculated from uncorrected sequence differences. Table S3B in [25] summarizes pairwise differences in concatenated segments of the mitochondrial *atp8*, *cob*, and *nad5* genes plus the control region; it is evident by inspection of the table that D/θ is even larger in this data set than in the *atp8* gene alone.

### Common Ravens Include Two Evolutionary Species; Chihuahuan Ravens May be a Third

Common ravens (*Corbus corax*) are distributed throughout most of the Northern Hemisphere. Their range overlaps almost completely with that of the Chihuahuan raven (*C. cryptoleucus*) in the southwestern United States and northern Mexico. Omland et al. [26] used mitochondrial gene sequences and nuclear microsatellite data to show that many of the common ravens in the western United States form a separate California clade from common ravens elsewhere in the US, Europe, and northern Asia. However, no apparent phenotypic differences separate these two clades. Chihuahuan ravens, which do show phenotypic differences from other ravens, formed a sister clade to the California clade with 73% bootstrap support. Additional analyses led Omland et al. to suggest that Old and New World ravens are in an intermediate stage of divergence, and that these two populations may have been completely separated in the past but are now merging to recreate a single species.

I downloaded all the raven *cob* sequences from GenBank and aligned them. I omitted the sequence from *Corvus corax* isolate CORA because it could not be aligned with the others. This left 100 sequences, plus five outgroups from among the corvids (*C. coronoidess, C. brachyrhynchos, C. albicolis, C. albus*, and a bat *Cynopherus horsfieldi*). These 100 sequences consisted of 8 from the Chihuahuan Raven *C. cryptoleucus*, 17 from *C. corax* Common Ravens of the California clade, and 75 from *C. corax* of the Holarctic clade as defined by [26]. The sequences were of very different lengths, so they were trimmed to 258 common sites (except that one of the trimmed sequences was missing one site at the 5′ end and another was missing one site at the 3′ end). Trimming most of the sites that are not present in all sequences avoids the problem of having pairwise sequence differences that are not strictly comparable because different sites may be evolving differently. The pairwise sequence differences between ingroup raven sequences were all 0.06 or smaller, so corrections for multiple hits would have been negligible and were not made.

**Figure 2:**
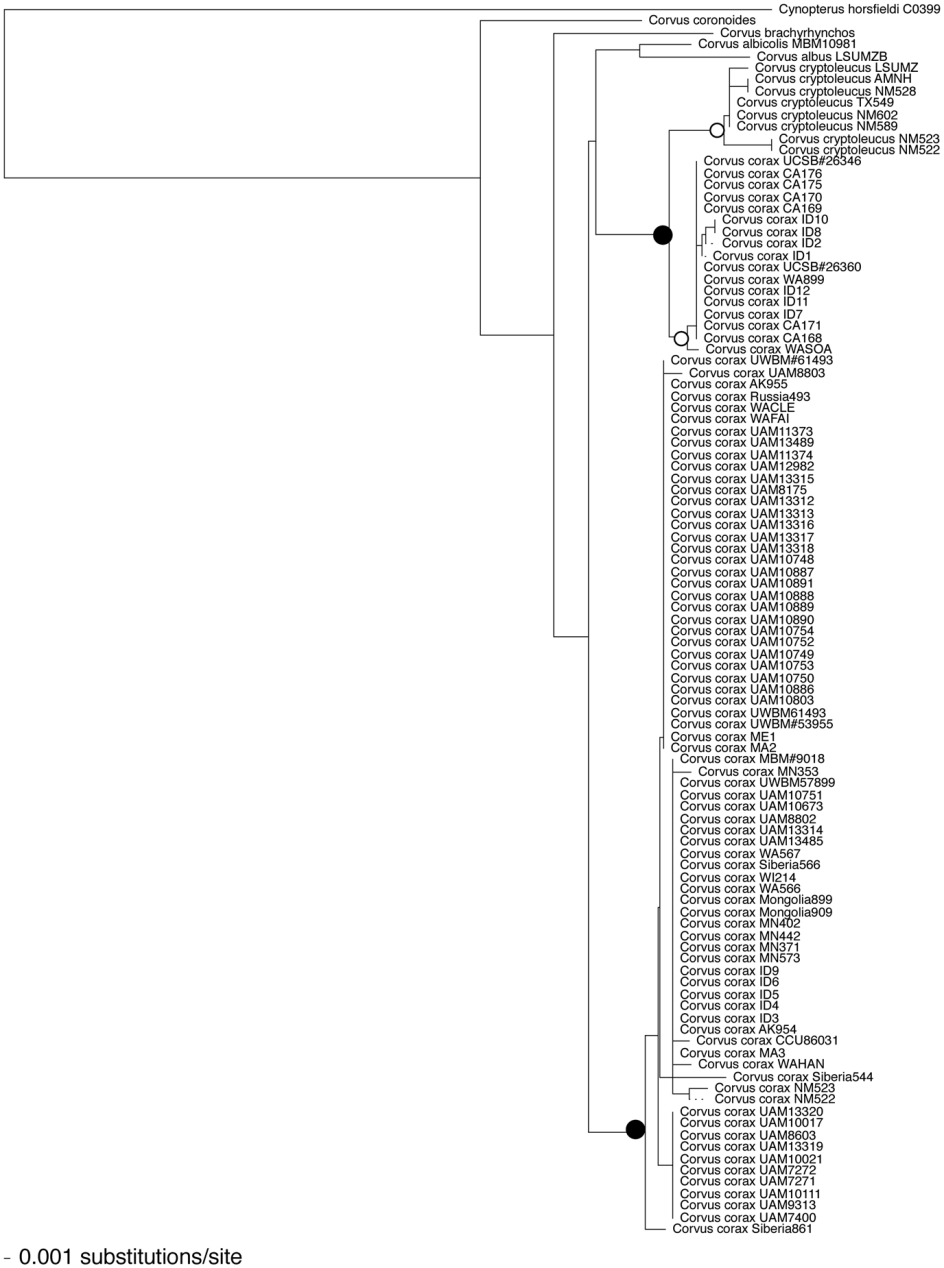
Neighbor-joining tree of raven cox1 sequences. Closed circles indicate clades that are different species, with D/θ>>4 (Table 1). Open circles indicate the Chihuahuan raven (C. cryptoleucus) and the Pacific clade of the common raven, which have D/θ<4 and thus are not different species by the criterion used here.

I made 1000 bootstrap replicas of Neighbor-Joining (NJ) trees of the sequences, rooted on the outgroup. The NJ tree is shown in Figure 2, with bootstrap support percentages on the nodes. In this tree the Chihuahan ravens formed a clade with 95% boostrap support while the California clade of the Common Raven had 73% support. These in turn formed a clade with 90% support, while the Holarctic Common Ravens form a clade with 87% support. This is the same tree topology found by [26] using *cob* plus the mitochondrial control region, and by Feldman and Omland [27] using sequences from the mitochondrial *cob*, *nad4*, and nuclear *cox1* genes and from a nuclear gene encoding a fibrinopeptide, all from a smaller subset of the raven individuals. Omland et al. [28] used mitochondrial control region sequences from Old World and New World ravens to obtain a maximum-likelihood tree in which all but one of the clades consisted only of either Old World or New World birds; however clades from these two geographic regions were intermixed.

An application of the D/θ ratio (summarized in Table 1) shows two species of ravens, *C. cryptoleucus* plus the California clade of *Corvus corax*, and the Holarctic clade *of C. cora*x. with D/θ = 16.4, and a probability of reciprocal monophyly >0.995. The first of these is in the process of speciation to separate the California clade of *C. corax* from the Chihuahuan raven *C. cryptoleucus*, or possibly fusion of the two species as suggested by [26]; the probability that these samples are from different clades is 0.93 and the D/θ ratio is 2.34. Thus the *cox1* sequences do not support the distinction between the Chihuahuan and common raven. Similarly, sequences of the mitochondrial control region gave a D/θ ratio of 2.6, again below the cutoff value of D/θ = 4.

**Table 1:** D/θ ratio calculations for Corvus cox1 sequences.

Clades	d	π	θ	D	D/θ	n1, n2	P(RM)
C. cryptoleucus + California clades vs. Holarctic clade	0.002	0.0021	0.0021	0.0349	16.4	75, 17	0.995
C. corax California clade vs. Holarctic clade	0.0012	0.0013	0.0013	0.0416	32.6	75, 17	0.0995
C. cryptoleucus vs. C. corax California clade	0.0076	0.0087	0.0088	0.0206	2.3	17, 7	0.93

### Arctic and Antarctic Populations of the Sea Butterfly *Limacina helicina* are Different Species

Sea Butterflies (or pteropods) are pelagic sea snails with aragonite shells which make them vulnerable to ocean acidification and consequently of interest as indicator species for acidification. Two subspecies with disparate ranges are recognized: *L. helicina helicina* from Arctic waters and *L. helicina antarctica* from Antarctic waters. Hunt et al. [29] obtained *cox1* sequences of 5 specimens from Arctic and 6 specimens from Antarctic waters. The Arctic and Antarctic specimens formed separate clades with 100% Bayesian and Neighbor-joining boostrap support. Hunt et al. concluded that “this degree of separation is sufficient for ordinal level taxonomic separation in other organisms and convincingly demonstrates that…the Arctic and Antarctic populations differ at least at the species level.”

**Figure 3:**
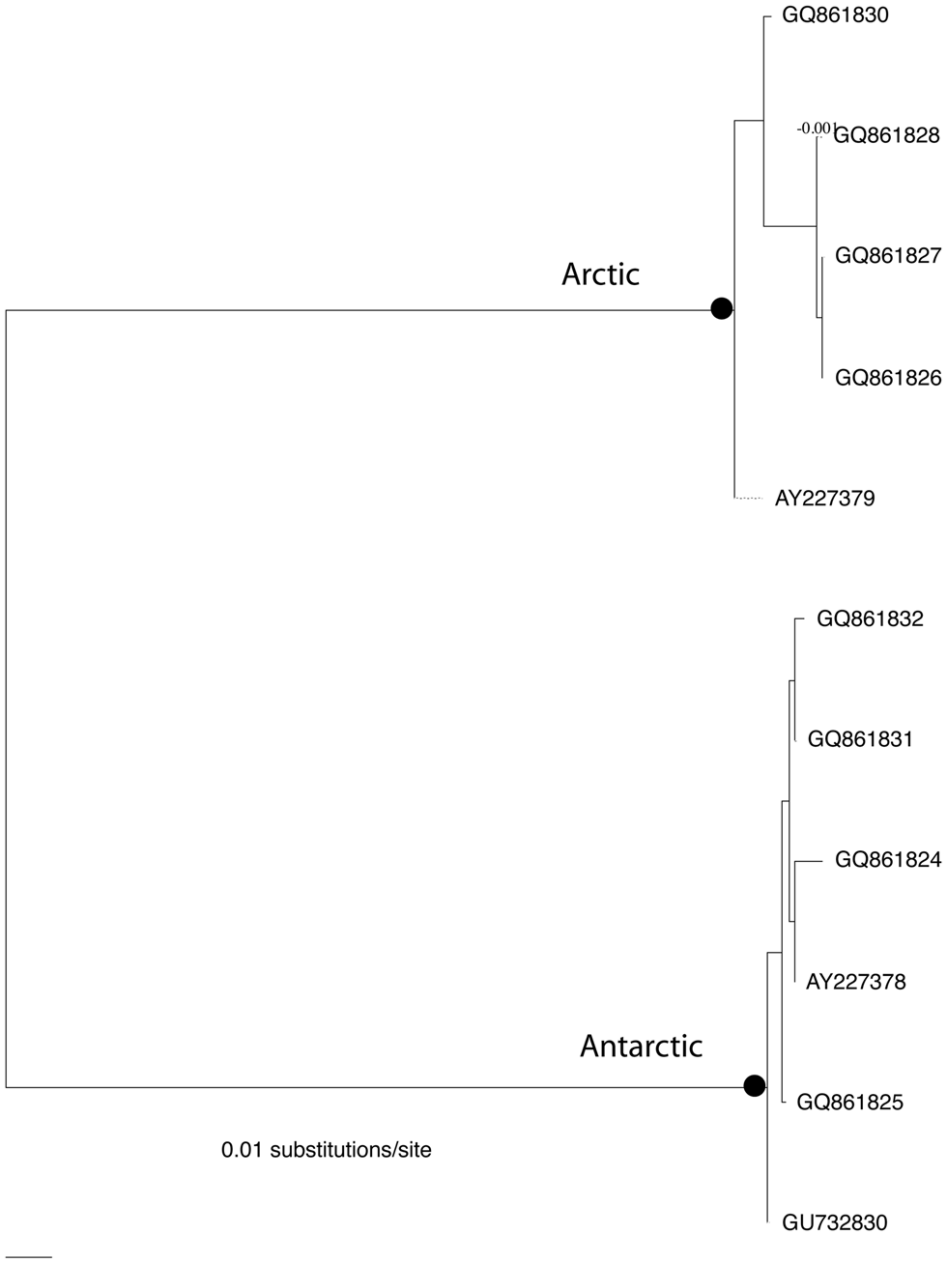
Pterapod cox1 tree, made with the neighbor-joining algorithm and the K81 model. Closed circles indicate clades that are different species, with D/θ>>4; Arctic and Antarctic clades are labelled.

I downloaded the *L. helicina* sequences from GenBank and trimmed them to the same length. Several sequences had a single missing internal base at one site; I left this site in the sequence. A neighbor-joining tree (Fig. 3) made with pairwise sequence differences corrected with the K81 (Kimura 81) model selected by ModelTest distances showed the same two well-separated clades reported by Hunt et al. [29], each supported by 1000/1000 bootstrap replicas. These clades differed by K = 0.3526 differences per site, corrected for multiple hits, and θ = 0.0092 and 0.0074 using uncorrected differences. The K/θ ratio using the larger θ was 38.5, which with the sample sizes 5 and 6 corresponded to a probability >>0.99 in Rosenberg's table. I conclude that the two clades are samples from different clades which are different evolutionary species. This confirms the conclusion of Hunt et al. that these subspecies are actually cryptic evolutionary species.

Both the Arctic and Antarctic clades include well-supported sub-clades (Fig. 3). The Antarctic clade consists of 2 singletons and a clade with 4 specimens supported by 70% of 1000 bootstraps with the K2P model. The K/θ ratio for either singleton and the clade was 1.37, corresponding to a probability of 0.4; this is much too low for these to be recognized as samples from different clades or different independently evolving populations and they must be considered a single species based on the *cox1* sequences.. The Arctic clade also includes 2 singletonons and a clade of 3 specimens (GQ861826, GQ861827, GQ861828) with 98% bootstrap support. The sequences of these 3 specimens are identical, which means that π and θ are estimated as zero and K/θ cannot be calculated. If one arbitrarily assumes that 1 of the 3 sequences differs from the other 2 at a single site, the K/θ value for this clade and the most closely related singleton (sequence AY227379) is 10.1, giving a probability greater than 99% that they represent separate evolutionary species. The K/θ value for this clade and the other singleton (GQ861830) was even larger at 14.2, so it is possible that the 5 specimens of *L. helicina helicina* from Arctic waters represent as many as three different species. However, given the *ad hoc* nature of the calculation of π and θ when d = 0 and the likely large uncertainty in the estimates, these data do not inspire confidence in splitting the Arctic population. Adding to the uncertainty, I have been unable to find the collection locations or information about the morphologies of the specimens that provided DNA sequences AY227378 and AY227379; thus it is possible that they were taken at a single site and are very closely related.

### Applying K/θ to Delimit Species in Liverworts

**Figure 4:**
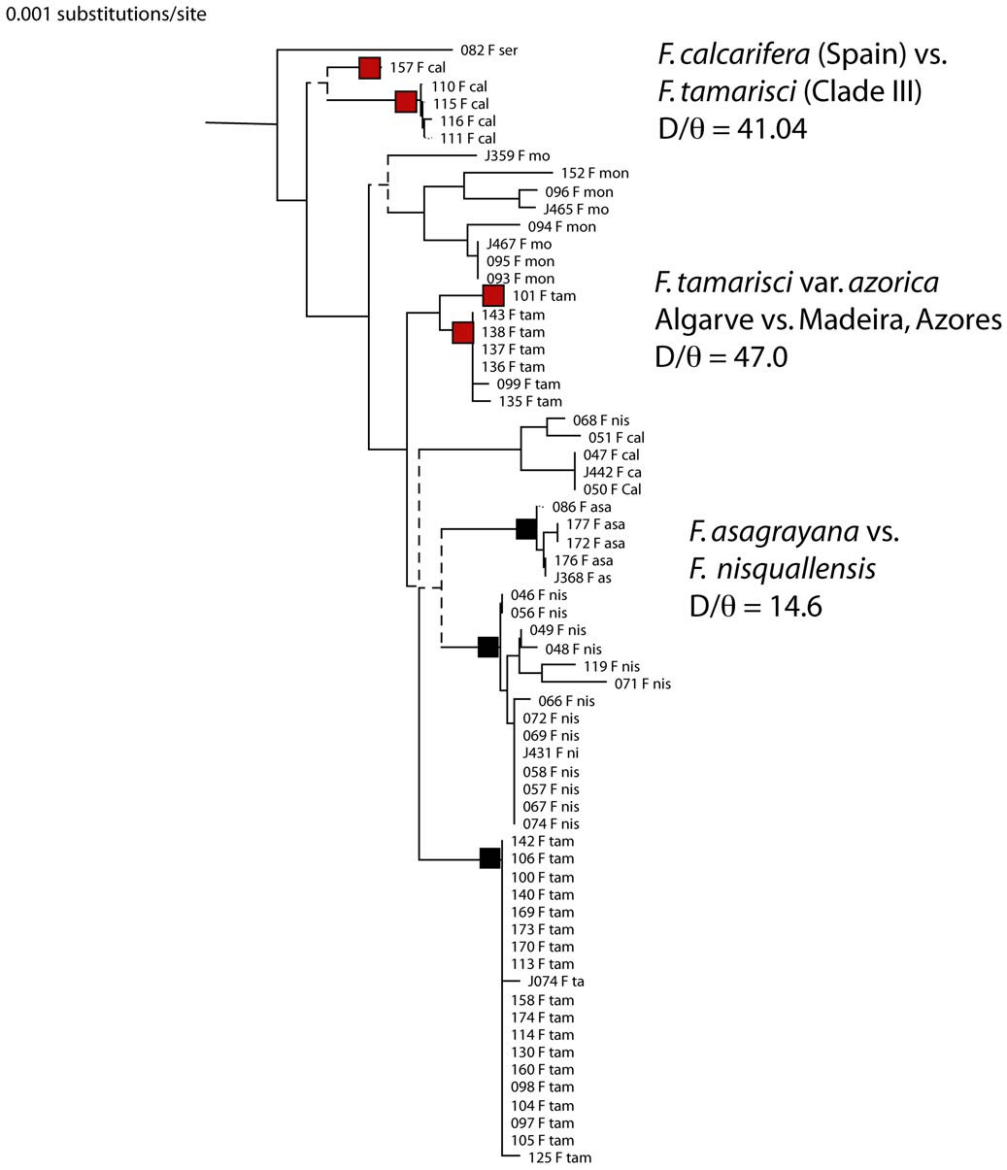
Neighbor-joining tree of partial sequences of the chloroplast genes AtpB and rbcL genes from liverworts. Sequence distances were small and not corrected for multiple hits. Dashed lines indicate clades supported by less than 500/1000 bootstrap replicas. Black squares indicate clades that are species identified by Heinrichs et al. [44] and verified using Kθ; red squares are species delimited by K/θ but not distinguished by Heinrichs et al. The probability of reciprocal monophyly in the populations from which the individuals were sampled was >0.95 in each case.

The use of K/θ to delimit species is obviously not limited to animals. As an example I used it to find evolutionary species in the liverwort species complex *Frullania tamarisci sensu lato*, using 564 base pairs of the chloroplast genes *AtpB* and *rbcL* sequenced by Heinrichs *et al.* [30]. Pacak and Szweykowska-Kulinksa [19] showed that chloroplast genes are inherited maternally in another species of liverwort. The neighbor-joining tree of uncorrected sequences (Fig. 4) reveals a number of clades, some of which were suggested to be different species by Heinrich *et al.*. I focused on testing four pairs of sister clades. All pairwise sequence differences are small so I used the uncorrected sequence differences D and calculated D/θ as a close approximation to K/θ

- Heinrichs et al. [30] evidently considered the four specimens of *Frullania tamarisci* clade III and the single specimen of *F. calcarifera* from Spain to be one species, but the populations are reciprocally monophyletic with probability >0.99 and D/θ = 41, clearly qualifying them as different evolutionary species.
- *F. tamarisci* var *azorica*, an individual from Algarve in Spain, and the individuals from Madeira in the Azores, are from different species. Heinrichs *et al.* considered these to be a single species, but the probability that the samples represent reciprocally monophyletic populations is >0.98 and D/θ is much greater than 4.
- *F. asagrayana* and *F. nisquallensis* are sister taxa in the neighbor-joining tree, but these two species and *F. tamarisci* form a polytomy in the bootstrapped tree. Consequently, I analyzed *F. asagrayana* vs. *F. nisquallensis*, finding P(RM)>0.99 and D/θ = 14.6. I then analyzed *F. tamarisci* with the clade with the smallest mean sequence divergence, *F. nisquallensis*, obtaining a probability of reciprocal monophyly >0.99 and D/θ = 6.5. From this it is obvious that *F. tamarisci* and *F. asagrayana* are also different species.

The first two cases illustrate that, when the sequence difference between two clades is very large, even very small samples suffice to recognize that they are samples from different clades and species. This is also apparent from an inspection of Figure 6 in [14].

## Discussion

### The K/θ Method

The K/θ method is a simple way to delimit species using DNA sequences. It does so by distinguishing transient gaps in genotypes due to stochastic effects from longer-lasting gaps due to natural selection or physical isolation that separate species. In a phylogenetic tree of gene sequences, it distinguishes between clades *within* species and clades that *are* species. In this respect it differs from phylogenetic species concepts that have been rightly criticized because they have no way to distinguish between species and varieties within a species.

### The K/θ Method and Traditional Systematics

As is common in the systematics literature, none of the papers cited in **Results** explicitly mention a species concept or model as guiding their efforts to assign sequences to one or more species. However, several different species criteria were mentioned explicitly or implied. In each case recprocal monophyly was used as part of the rationale for splitting species; in the cases of the clouded leopards, pterapods, and liverworts this was coupled with large sequence differences between the putative species, similar to differences between species in other groups. A major difference between these approaches to species delimitation and mine is that the K/θ method actually measures the sequence difference between putative species and compares it to differences within species, as opposed to relying on intuition to decide when branches are long enough to separate species.

### Sampling

It cannot be overemphasized that the sequences are from individuals that are sampled from a species, or from several species, and that the number of specimens in the sample is very small compared to the population size. In fact this is the case for all species delimitation [31]. An obvious exception is the case of endangered groups with extremely small population sizes, such that it is possible at least in principle to sample a large proportion of the individuals; but even if one had sequences from all individuals in a group of one or more species, it would still be necessary to use something like the K/θ ratio to delimit species.

An important difference between the K/θ method and most other approaches to delimiting species is that it explicitly recognizes that species are inferences based on finite samples of individuals, and as such is subject to sampling error. The K/θ method deals with that potential error by estimating the probability that a set of specimens came from one species or two. The K/θ ratio can be used to delimit species without reference to the sample sizes by using the “4× rule”, which recognizes that for all but the smallest sample sizes, when K/θ≥4, the probability that the sequences are from different species is ≥0.95 [4], [5], [6]. As can be seen in Rosenberg's Figure 6 [14], when the number of individuals in the sample is very large and they into two different clades, one can infer that the sampled individuals came from two reciprocally monophyletic groups even when K/θ ratios much less than 4. However, this does not mean that those clades belong to different species, as opposed to transient clusters formed by stochastic processes, with probability ≥0.95; to infer this, we need K/θ≥4.

The K/θ method also takes into account another aspect of sampling, namely the finite number of sites in the gene sequences. It does so by requiring that the clades are seen in a phylogenetic tree and are well-supported by bootstrapping before we can infer that they came from ≥2 different species. Boostrapping could be replaced by a high Bayesian probability or other methods that attach a probability to clades.

Of course, the K/θ method does not take into account the possibility that the specimens in the sample may not be representative of the populations from which they came; for example, the sample should not consist entirely of one set of siblings, because they will be more closely related than a random sample and will greatly underestimate θ. However, no method of species delimitation can compensate for inadequate sampling. The only way to determine if samples adequately represent the populations is by increasing the sample sizes and the number of locations and habitats sampled to see if the increased sampling either lumps or splits the species found in the initial sample.

### Singletons

Lim *et al.* [32] noted that many species descriptions are based on a single specimen, and discussed the difficulties posed by singletons for discriminating species. Obviously the K/θ ratio cannot be used to determine whether two singleton sequences represent different species when they are sisters in the phylogenetic tree, because there is no way to estimate θ for either candidate species from this pair of samples. The case of a singleton that is sister to a clade of two or more specimens is somewhat different, because in that case the clade can be used to estimate θ. Of course in doing so we run the risk that one or more new specimens will be discovered that will form a clade with the singleton and result in K/θ<4, requiring lumping of the two species. But as noted above, this is possible with any method of species delimitation; systematics will always be a work in progress for most organisms.

### Species Splitting and Lumping

The phrase “species delimitation” may seem to imply that the K/θ ratio is used only to decide whether a sample belongs to one species or to two or more species. However, the K/θ ratio can be used either to determine whether an already-described species should be split into two or more evolutionary species, or whether two or or more described species should be lumped because they are actually a single evolutionary species. In the cases analyzed here, there are several in which the K/θ ratio indicates that species should be split (clouded leopards, sea butterflies, liverworts I *tamarisci* var. *azorica*). In contrast, *F. asagrayana* and *F. nisquallensis* are validated as separate species, as are *Frullania tamarisci* clade III and the single specimen of *Frullania calcarifera* from Spain.

The application of molecular data often results in splitting species; apparently many evolutionary species are cryptic. It is probably less likely that two species distinguished by the K/θ method will be joined by new data. This would require finding new specimens that joined the two species into a single clade with a new value of πthat is K times greater, a value that would often be well outside the usual range of nucleotide diversities for that group of organisms.

### Comparison of the K/θ Method to Other Delimitation Methods

The primary purpose of this paper is to explain and illustrate the application of the K/θ ratio to species delimitation in sexual organisms, and a comprehensive review of other methods and comparison to K/θ is beyond the scope of the paper. However, a few general comments are appropriate to put K/θ in context.

The K/θ method is designed to detect a significant gap between pairwise sequence differences that are in different species. Traditional barcoding and the Automatic Barcode Gap Discovery (ABDG; [33]) program also look for such a gap, but the gap is not based on population genetic theory. Instead, these methods look for a gap in data from traditional species and then use the presence or absence of such a gap in new data to delimit species. For this purpose the traditional species are assumed to be well defined; this is not always true and the K/θ method makes no such assumption. Moreover, the size of the gap is estimated from pooled data of many species pairs, which can obscure gaps when different pairs have gaps at different positions in the frequency distribution. In contrast, the K/θ method looks only at sister clades. ABDG has been directly compared to K/θ and GMYC, and shown to significantly underestimate species diversity [13].

M [34] has some similarity to K/θ. Starting with an ulrametric tree, M is the ratio of the distance from a node that is the base of a putative species to the tips, divided by the distance between the node and the root. From this is calculated the probability that the sequences came from a single panmictic species. When θ is small, M is approximately θ/(1/2)K, or the reciprocal of K/2θ. A striking difference between M and the K/θ method is that M attempts to show whether a single clade is one species or more than one species, rather than asking whether a pair of sister clades represent different species. This is probably the basis of the unsolved problem acknowledged by the authors [34], that the test result for one clade changes the outcome for another.

The generalized mixed yule coalescent (GMYC) method of Barraclough and collaborators [12] uses maximum likelihood to identify the point on an ultrametric tree where the branching changes from a slow rate reflecting speciation events to a much faster rate reflecting generations of reproduction. Presumably, that point will be clearly defined if there is a barcode gap. GMYC has been directly compared to K/θ using several different databases, and it usually identifies the same species [6], [7], [13], [35]. However, GMYC encounters difficulties in dealing with identical sequences and with very small numbers of sequences (Timothy Barraclough, personal communication).

A number of species delimitation methods have been developed to use with multiple nuclear genes. Although some of these can delimit species at an earlier stage than the K/θ method, the requirement for multiple genes greatly increases the cost. The coalescent-based methods SpedeSTEM, BPP, and ABC are reviewed and compared in [36]. These methods require that samples be assigned to putative species at the beginning; if the initial assignment is incorrect, it can cause errors in the final result. An advantage is that they can potentially detect speciation events before genes become reciprocally monophyletic. For example, SpedeSTEM [37] can validate evolutionary lineages separated by ≥0.5N_e_ generations, which corresponds to 2N_e_ generations for organelle genes. However, this level of resolution requires sequence data from ≥5 nuclear loci.

Several methods use multiple nuclear genes to jointly estimate species assignments and trees without assuming *a priori* species. KC delimitation finds the species tree that best fits the gene trees, while nonparametric delimitation is based on the principle that branches where many different genes have the same topology are likely to be between, rather than within, species [38]. Gaussian clustering [39] delimits species with dominant or codominant allelic data. Structurama [40] requires that sequence data be converted to alleles, which are assigned to populations (species) so as to minimize linkage disequilibrium and maximize Hardy-Weinberg equilibrium. Structurama is able to delimit species diverged as recently as 1.5N_e_ generations with ≥10 loci. However, this level of resolution requires ≥5 loci.

### Limitations of the K/θ Method

The K/θ method can fail in cases of very recent speciation where two populations have become completely separated (complete reproductive, physical, or ecological isolation) but even the early-segregating mitochondrial or chloroplast genes have not yet become reciprocally monophyletic. Even if the populations are reciprocal monophyletic, K may not much larger than θ when speciation is rapid. However, most methods of species delimitation are likely to fail in these situations; some exceptions are mentioned in the preceding paragraph.

### The Choice of Genes

In principle, the K/θ method can be used with nuclear genes as well as genes from mitochondria or chloroplasts, but on average the organelle genes will differentiate species that are more recently diverged. Obviously if speciation is sympatric, involving reproductive isolation determined by one or a few genes, it would be better to use K/θ with those genes. However, identifying genes responsible for speciation is difficult and expensive, and has been done in only a few cases. Similarly, although it is likely that some nuclear genes will, by chance, complete stochastic sorting to become reciprocally monophyletic earlier than organelle genes, it is effectively impossible to know what genes those are without testing most or all of the nuclear genome.

Male migration with female philopatry would not be detected using *cox1* or other mitochondrial genes, or with chloroplast genes in plants with exclusively or almost exclusively maternal inheritance. In such cases, the method described here would detect speciation in some cases where it is, as yet, incomplete. Where traditional taxonomy recognizes two populations as distinct species even though they are still exchanging mitochondrial genes, the exchange of mitochondrial genes is called mitochondrial introgression. However, populations that are exchanging genes at an appreciable level are unlikely to be evolving independently and hence should not be assigned to different species under the evolutionary species model. For an entry to the recent literature on this subject, see [41], [42] and references therein.

This is not likely to be a problem for the clouded leopards and ravens considered in this paper. The behavior of the secretive clouded leopards is not well known, but a radio telemetry study found that the territories of males and females are similar in size and overlap substantially [43]. According to Bernd Heinrich ([44] and personal communication), it is very unlikely that there are sex differences in raven dispersal. This is based on their social behavior in which juveniles move around in groups but later as adults form monogamous pairs that remain together in one location where they nest.

It should be noted that some plants inherit mitochondrial and/or chloroplast genes paternally, so that it is the preferential migration of females, not males, that would be misleading. Also in some organisms these genes are inherited biparentally at least occasionally, which would partially alleviate the problem.

### One Gene or Many?

If one believes that all, or some percentage, of nuclear genes must complete soring before two lineages are considered to be different species, then multiple nuclear genes would be better. But this is not required by the evolutionary species concept. Consider that the demonstration of reproductive isolation would prove independent evolution, and is widely recognized as sufficient evidence even though it is possible that a single nuclear gene locus is responsible.

Moreover, using a mitochondrial or chloroplast gene has the advantage that one knows *a prioi* that these genes detect an earlier stage of speciation than most nuclear genes because the coalescence time is as little as ¼ as long as the average for nuclear genes. For the same reason, using one or more nuclear genes *in addition to* an organelle gene is most likely to cause one to detect speciation at a later stage. One cannot even know how much later, because different nuclear genes are likely to be unlinked and have different coalescence times just by chance. In short, adding a nuclear gene would be likely to obviate the advantages of using the organelle genes. An exception would be if one could identify the gene(s) responsible for speciation, usually by reproductive isolation, but this is expensive and rarely done. Also as noted above, there are some methods that use multiple nuclear genes to detect very early stages of speciation; perhaps some variant of these methods could incorporate organelle genes together with the information that we that the expected coalescence time for these genes is much shorter than that of the nuclear genes.

### Which θ to Use?

In most cases the two sister clades being tested will have different values of θ. In the examples presented here I used the larger value of θ to calculate K/θ. This is the conservative choice, in the sense that it makes K/θ smaller and therefore is more likely reject the hypothesis that the clades are samples from different species.

#### Using K/θ with Singletons

Lim et al. [32] noted that many species are represented by only a single specimen in museums and other collections, and discussed the problems that these singletons represent for several methods of species delimitation. The Kθ method can define a species with at least 95% probability based on only one single specimen, provided the phylogenetic tree shows it to be the sister to a clade containing at least 5 specimens and separated from it by K/θ≥4. Higher K/θ ratios can differentiate species with fewer specimens; for example, a singleton and a clade of 2 specimens separated by K/θ = 4.2 have a probability of 0.9506 of coming from different populations. Obviously in these cases θ can only be estimated from the sister clade.

#### The Future: Using DNA Alone in New Species Descriptions and Speciation Studies

I have not attempted to write formal new species descriptions of any of the cryptic species detected with the K/θ method in this paper, preferring to leave that to the original authors and/or professional systematists. I agree with the arguments of Cook et al. [45] and others that there is no impediment to doing so in theory or in the zoological or botanical codes of nomenclature. But there are some practical problems. One is that if a morphological species consists of two or more cryptic species distinguishable only by DNA sequences, it may not be feasible to use sequence data to distinguish them in some future studies. Another is that it will often be difficult or impossible to decide whether a cryptic species corresponds to a published species description, which will usually not include DNA sequences. I will address these problems in a future paper; neither is insurmountable.

Finally, a K/θ ratio less than 4 could in principle be applied to two populations that are known or suspected to be in the process of speciation in order to determine how far the process speciation has progressed.
